# CO-MORBID DEMENTIA AND CANCER THERAPY DECISION-MAKING: A SCOPING REVIEW

**DOI:** 10.1093/geroni/igad104.0654

**Published:** 2023-12-21

**Authors:** Sean Halpin, Gabriel Alain, Hui Zhao, Mackenzie Fowler, Qiuyang Zhang, Tamara Cadet, Minzhi Ye, Jessica Krok-Schoen

**Affiliations:** RTI, Decatur, Georgia, United States; The Ohio State University, College of Medicine, Columbus, Ohio, United States; James Madison University, Harrisonburg, Virginia, United States; University of Alabama at Birmingham, Birmingham, Alabama, United States; Tulane University School of Medicine, New Orleans, Louisiana, United States; The University of Pennsylvania, Philadelphia, Pennsylvania, United States; Kent State University, Kent, Ohio, United States; The Ohio State University, Columbus, Ohio, United States

## Abstract

Co-morbid dementia complicates cancer therapy decision-making in older adults. Our scoping review examined cancer therapy decision-making among older persons with co-morbid cancer and dementia and their caregivers. Pubmed, Embase, CINAHL, PsycINFO, Cochrane Library, Scopus, and Web of Science were searched from 2018-2022, resulting in 8,654 articles screened, with eight meeting the inclusion criteria. Most studies were conducted in the UK (89%) and reported homogeneity in race and geography. Breast (56%) and prostate (45%, with one reported stage) were the most frequent cancers reported. Five studies (56%) reported multiple types of dementia, with two (22%) indicating stages. The studies indicated that communication between patients, caregivers, and clinical teams might alleviate stress caused by worsening health prospects. Future research should consider diverse racial, geographic, cancer types, dementia types, and healthcare stages to improve understanding of these decision-making processes.

